# Therapeutic Efficacy of Percutaneous Radiofrequency Ablation versus Microwave Ablation for Hepatocellular Carcinoma

**DOI:** 10.1371/journal.pone.0076119

**Published:** 2013-10-17

**Authors:** Lei Zhang, Neng Wang, Qiang Shen, Wen Cheng, Guo-Jun Qian

**Affiliations:** 1 Department of Ultrasound, Harbin Medical University Cancer Hospital, Harbin, China; 2 Department of Minimal Invasion, Ward 1, The Second Military Medical University Eastern Hepatobiliary Surgery Hospital, Shanghai, China; Northwestern University Feinberg School of Medicine, United States of America

## Abstract

The aim of this study was to investigate the therapeutic efficacy of percutaneous radiofrequency (RF) ablation versus microwave (MW) ablation for hepatocellular carcinoma (HCC) measuring ≤5 cm in greatest diameter. From January 2006 to December 2006, 78 patients had undergone RF ablation whereas 77 had undergone MW ablation. Complete ablation (CA), local tumour progression (LTP) and distant recurrence (DR) were compared. The overall survival curves were calculated with the Kaplan-Meier technique and compared with the log-rank test. The CA rate was 83.4% (78/93) for RF ablation and 86.7%(91/105 for MW ablation. The LTP rate was 11.8% (11/93) for RF ablation and 10.5% (11/105) for MW ablation. DR was found in 51 (65.4%) in the RF ablation and 62 (80.5%) in the MW ablation. There was no significant difference in the 1-, 3-, and 5-year overall survival rates (P = 0.780) and the 1-, 3-, and 5-year disease-free survival rates (P = 0.123) between RF and MW ablation. At subgroup analyses, for patients with tumors ≤3.0 cm, there was no significant difference in the 1-, 3-, and 5-year overall survival rates (P = 0.067) and the corresponding disease-free survival rates(P = 0.849). For patients with tumor diameters of 3.1–5.0 cm, the 1-, 3-, and 5-year overall survival rates were 87.1%, 61.3%, and 40.1% for RF ablation and 85.4%, 36.6%, and 22% for MW ablation, with no significant difference (P = 0.068). The corresponding disease-free survival rates were 74.2%, 54.8%, and 45.2% for the RF ablation group and 53.3%, 26.8%, and 17.1% for the MW ablation group. The disease-free survival curve for the RF ablation group was significantly better than that for the MW ablation group (P = 0.018). RF ablation and MW ablation are both effective methods in treating hepatocellular carcinomas, with no significant differences in CA, LTP, DR, and overall survival.

## Introduction

Hepatocellular carcinoma (HCC) is the sixth most common type of cancer and the third leading cause of cancer-related death [1]. Hepatectomy offers the best outcomes for patients with HCC [2]–[3]. Unfortunately, there are less than 30% of cases are amenable to hepatectomy at the time of diagnosis due to advanced tumor stage and underlying liver cirrhosis [4]–[5]. When hepatectomy options are precluded, image-guided tumor ablation therapy is recommended as the most appropriate therapeutic choice and is considered a potentially curative treatment in properly selected candidates [4], [6]. In the past two decades, thermal ablation therapy by using energy sources has been increasingly accepted due to the advantages of greater capacity to devitalize HCC with fewer treatment sessions [7]–[8]. Among them radio-frequency (RF) ablation and microwave (MW) ablation are the most commonly used modalities. Radiofrequency (RF) ablation has been considered to be the most common thermal ablation modality worldwide for early stage HCC in patients, with 80–95% complete tumour necrosis and 33–57% 5-year survival [9]. MW ablation, which generates an electromagnetic field in the tissue and causes rapid and homogeneous rotation of molecules in order to damage the tumour thermally [10], is common in China and Japan; however, it has been introduced to western countries recently [11]–[12]. Studies in which MW ablation was used to treat HCC ≤5 cm have reported 29–68.6% 5-year survival [13]–[14].

Studies on comparing HCCs treated by these two thermal ablative techniques have been carried out by several groups. Randomized control trial found no significant differences in local tumor control, complication rates, and long-term survival for small HCC measuring ≤4 cm between RF ablation and MW ablation [15]. To our knowledge, however, a few studies have been performed to compare the two modalities for the treatment of HCCs measuring ≤5 cm in greatest diameter. The aim of this study was to investigate the therapeutic efficacy of percutaneous RF ablation versus MW ablation for hepatocellular carcinoma measuring ≤5 cm in greatest diameter. In this study, HCCs measuring ≤5 cm in greatest diameter was defined as a solitary HCC lesion of 5 cm in greatest diameter or smaller, or three or fewer multiple HCC lesions with a greatest diameter of 3 cm or less.

## Materials and Methods

### 1. Ethics statement

All examinations and treatments performed at our Institution were performed in accordance with the Helsinki Declaration and were approved by the Eastern Hepatobiliary Surgery Hospital Ethics Committee in the Second Military Medical University, Shanghai, China. Written informed consent was obtained from all patients enrolled in the study. This was a retrospective study of prospectively collected data.

### 2. Patients

A total of 155 eligible patients with conclusive diagnoses of HCC underwent precutaneous ablation were enrolled in this retrospective study from January 2006 to December 2006. Among these patients, 78 (with 97 tumours) had undergone RF ablation whereas 77 patients (with 105 tumours) had undergone MW ablation. According to the sequence of the patients visit to the hospital, RF or MW ablation was selected for them without randomization. The patients' baseline characteristics are summarised in Table 1.

**Table 1:** Baseline Characteristics of the Study Patients.

Parameter	RF Ablation Group (n = 78)	MW Ablation Group(n = 77)	P value
M/F ratio	64∶14	67∶10	NS
Age (y)*	54±10.5(30–80)	54±9.5 (26–76)	NS
Tumor size* †			NS
≤3 cm	47	36	
3.1–5.0 cm	31	41	
a -fetoprotein level			NS
≤400 ng/mL	63	56	
>400 ng/mL	15	21	
Hepatitis B surface antigen			NS
Positive	75	71	
Negative	3	6	
No. of tumors			P<0.05
1	63	56	
2	11	14	
3	4	7	
Child-Pugh class			NS
A	78	77	

* Data are means ± SD. Numbers in parentheses are ranges.

† The mean tumor size was 2.3 cm±0.4 (0.8–5.0 cm) in the RF ablation group and 2.2 cm±0.4 (0.9–5.0 cm) in the MW ablation group.

NS: Not significant.

Diagnosis of all HCCs was based on the typical findings of either triphasic contrast-enhanced CT (CECT) or magnetic resonance imaging (MRI). The classic imaging profile associated with an HCC lesion is characterized by intense arterial enhancement or uptake followed by contrast washout in the delayed venous phase [6].

The criteria for use of precutaneous ablation in HCC were as follows: a solitary HCC lesion of 5 cm in greatest diameter or smaller; three or fewer multiple HCC lesions with a greatest diameter of 3 cm or less; no radiologic evidence of vascular invasion and extrahepatic metastases; index tumours with no prior treatment; liver function classed as Child-Pugh class A; prothrombin time of less than 22 s and platelet count higher than 45 cells×10^9^/L; no history of ascites refractory to diuretics, variceal bleeding, or encephalopathy.

### 3. Percutaneous Ablation Procedures

Pethidine 100 mg and anisodamine hydrochloride (654-2) 10 mg were given by intramuscular injection as the basal anesthesia before operation. All ablation procedures were performed under local anesthesia with 1% lidocaine from the insertion site in the skin down to the peritoneum along the planned puncture track, and conscious analgesia-sedation was induced by intravenous administration of 0.1 mg of Tramadol (SanJiu Pharmaceutical Ltd., Zhejiang, China). The skin of the insertion site was incised with a small Lancet. Real-time ultrasound (EUS-405, Hitachi, Japan) was used for the guidance and monitoring of ablation procedures, equipped with a 4–8 MHz convex probe. Patient's posture was changed according to the preoperative tumor localization detection under real-time ultrasound. The aim of the treatments was to destroy the entire tumor with a safety margin of 0.5–1.0 cm. Vital signs and oxygen saturation were also monitored during the procedure.

#### 3.1 Precutaneous RF ablation

An Elektrotom HiTT ablation system (Berchtold, Medizinelektronik, Germany) was used in this study. This system is composed of a 375-kHz computer-assisted RF generator, a 15-gauge electrode (Berchtold, Tuttlingen, Germany) and two dispersive pads to deliver therapeutic energy directly to the tumour. The electrode has a 15-mm-long electrode tip with lateral aperture but no stomidium, with 15–20 cm in length. The 15-gauge electrode was introduced into the tumor, with the tip at the bottom of the tumor. We set the RF generator output power at 60 w, with the duration of 6–20 min for each single application, thus keeping the machine working at low impedance (100–350Ω). During the procedure, continuous perfusion of physiological saline through the lateral aperture was administered automatically by means of a syringe pump (Pilot C, Fresenius Medical Care, Alzenau, Germany) linked to the radiofrequency generator. The infusion speed was administered at a base rate of 20 ml/10 min. and physiological saline infusion reach 40∼60 ml in each patient. For tumors ≤3.0 cm in greatest dimension, single applicator position with 1–2 durations was adopted. For tumors >3.0 cm in greatest dimension, different applicator positions were adopted to create overlapping coagulation zones [16]. At the end of the procedure, the needle track was cauterized for 15 sec to prevent possible tumor seeding or bleeding.

#### 3.2 Precutaneous MW ablation

A FORSEATM MW ablation system (Qinghai Microwave Electronic Institute, Nanjing, China) was used in this study. This system is composed of two MTC-3 MW microwave generators, two flexible coaxial cables and two cooled-shaft antennas. The generator is capable of producing 1–100 W of power at 2450 MHz. The cooled shaft antenna has a 14-gauge shaft coated with Teflon to prevent adhesion. Inside the antenna shaft, there are dual channels through which saline solution of 4°C is circulated by a pump to cool the shaft continuously. A BT01-100 steady-flow pump (LanGe-Pump, LanGe Steady Flow Pump Corp., Baoding, China) was used to pump the chilled saline solution circulating within channels of the antenna shaft at 40 ml/min.

Under real-time US guidance, the antenna was percutaneously introduced into the tumors with the tip placed in the deepest part of the nodule. For tumors ≤3.0 cm in greatest dimension, Single-application MWA was performed in an automatic mode for 8 min with 80 W. For tumors >3 cm, overlapping ablations were conducted as necessary. To prevent possible tumor seeding or bleeding, the needle track was cauterized for 15 sec when withdrawing the antenna.

### 4. Laboratory data

Major biochemical laboratory and haematological variables including liver function in terms of total bilirubin (TB), aspartate aminotransferase (AST), alanine aminotransferase (ALT) and albumin (ALB) levels ,and complete blood cell counts were tested and compared 2–4 days before and 48 h after ablation.

### 5. Effectiveness and follow-up

**Figure 1:**
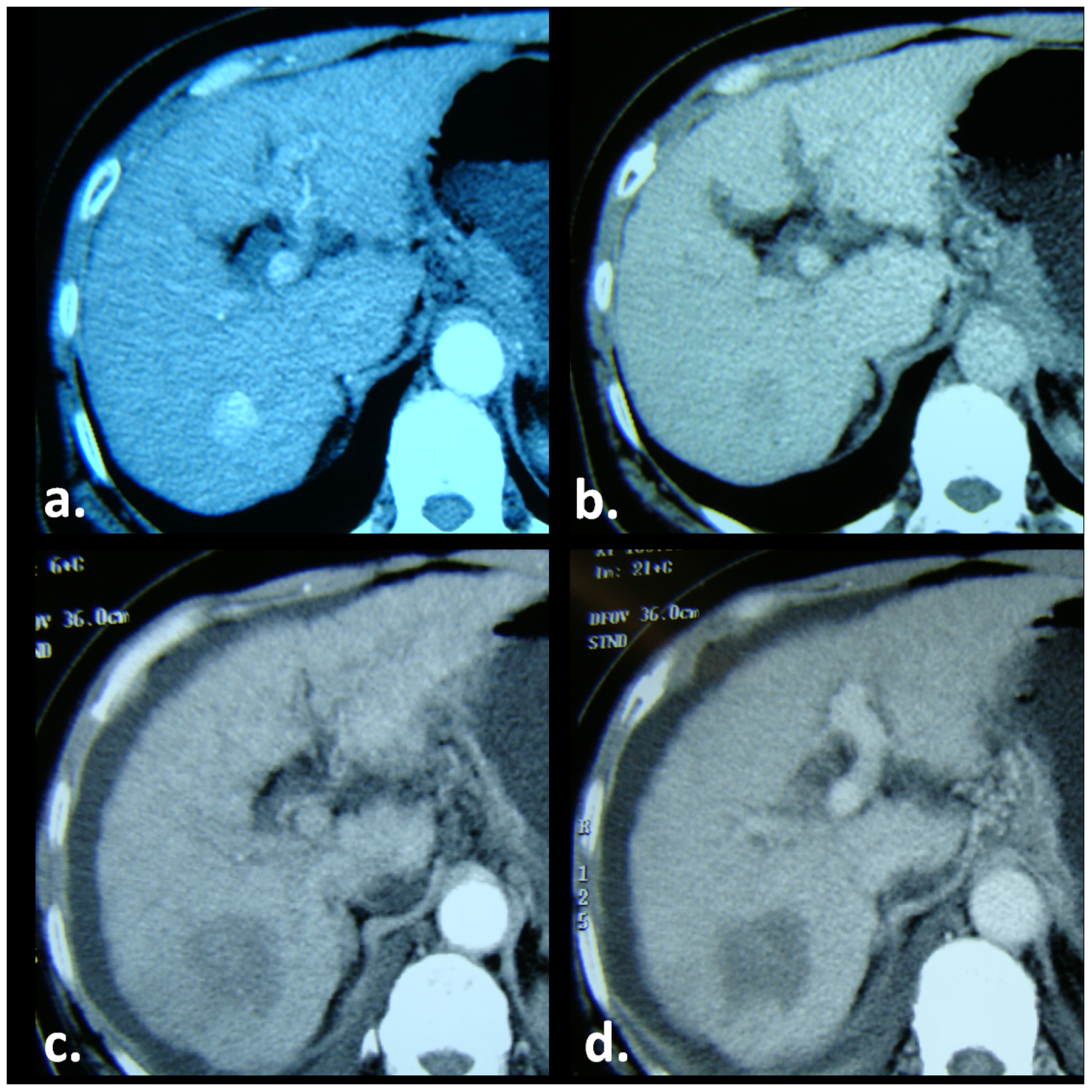
MW ablation of a HCC in a 50-year-old man with hepatitis B–related liver cirrhosis. (a) Arterial and (b) portal venous phase pretreatment CT images show tumor as a small 22-mm intense arterial enhancement nodule on a, with contrast washout on b. (c) Arterial and (d) portal venous phase CT images obtained 1 month after treatment show tumor has been replaced by nonenhancment ablation zone with a diameter greater than that of treated tumor. Findings are consistent with complete ablation (CA).

**Figure 2:**
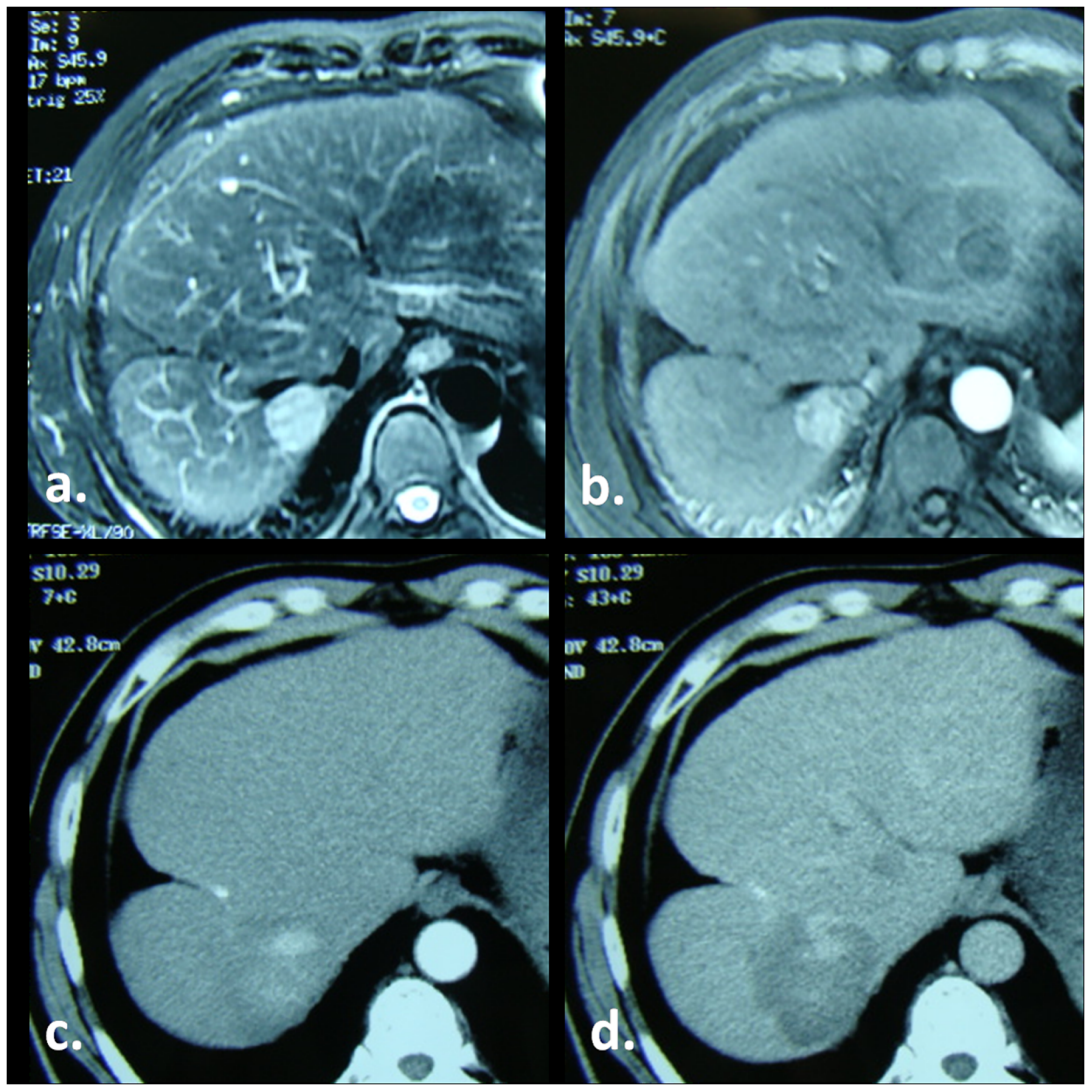
RF ablation of a HCC in a 64-year-old man with hepatitis B–related liver cirrhosis. (a) T2-weighted and (b) Arterial phase pretreatment MR images show tumor as a small 27-mm hyperintense nodule on a, with intense arterial enhancement on b. (c) Arterial and (d) portal venous phase CT images obtained 1 month after treatment show an irregular contrast enhancement found inside the ablation zone Findings are consistent with incomplete ablation (IA).

Technique effectiveness was evaluated by comparing contrast-enhanced CT (CECT) or CEMRI images performed 2–8 days before with those obtained 1 month after the treatment. On the basis of our previously published study [9] and our experience, complete ablation (CA) was defined as uniform low attenuation on CT without enhancement in the ablation zone with a diameter greater than that of treated tumor (Fig. 1). Incomplete ablation (IA) was defined as any irregular contrast enhancement found inside or beside the ablation zone (Fig. 2). Additional RF or MW ablation was performed for tumors with IA. Any ablation-related side effect or complication was documented. The definition of major complication is an event that leads to substantial morbidity and disability, increasing the level of care, or results in hospital admission or substantially lengthened hospital stay [17]. All other complications are considered minor. Side effects are expected undesired consequences of the procedure that, although occurring frequently, rarely if ever result in substantial morbidity. These include pain, the postablation syndrome, asymptomatic pleural effusions, and minimal asymptomatic perihepatic (or renal) fluid or blood collections seen at imaging [17].

In our department, primary HCC with CA were regularly followed up in the outpatient clinic with every 3 months for the first 2 years, and every 6 months thereafter from 3 to 5 years. Liver function tests and a-fetoprotein level was measured. Contrast-enhanced CT/MRI are considered the ‘gold standards’ and are commonly to evaluate the efficacy of thermal ablation. Local tumor progression (LTP) was defined as the appearance of tumor enhancement inside or adjacent to the ablated nodule, and distant recurrence (DR) was defined as new presence of intra-hepatic HCC [12]. For patients with LTP or DR who still met the precutaneous ablation inclusion criteria, RF or MW ablation was recommended. If patients did not meet the inclusion criteria, TACE, or conservative treatment was recommended.

The primary endpoint was 5-year survival, the secondary end point was disease-free survival. Other endpoints included laboratory variables, complications, CA, LTP and DR. Survival time was defined as the date of the operation to the date of the death or the last follow-up. The last follow-up date considered was 30/05/2012.

### 6. Statistical analysis

Continuous results were reported as the mean ± SD. Student-t test was used for comparison of the liver function and blood count 2–4days before and 48 h after ablation. Chi-square test or Fisher exact probability was used for comparing the rates of CA, LTP, DR, and complications between RF and MW ablation. The overall survival curves were calculated with the Kaplan-Meier technique and compared with the log-rank test. Survival curve was counted by month. A two-tailed P-value<0.05 was considered statistically significant. Data analyses were performed using JMP-8 software (SAS Institute, Cary, USA).

## Results

### 1. Patient groups

The patients' baseline characteristics are summarised in Table 1. There was no significant difference between the two groups in sex, age, tumor size, a-fetoprotein level, hepatitis B surface antigen, and Child-Pugh class. However, differences between the two groups with respect to No. of tumors in each patient were statistically significant (P<0.05).

For the 78 patients in the RF ablation group, 93 sessions of RF ablation were performed. A single RF ablation session was performed in 63 patients, and fifteen RF ablation sessions were performed in fifteen patients for tumors with IA 1 month after the first session. For the 77 patients in the MW ablation group, 91 sessions of MW ablation were performed. A single MW ablation session was performed in 63 patients, and fourteen MW ablation sessions were performed in fourteen patients for tumors with IA 1 month after the first session. All of the tumors with IA got fully ablations after additional one session of RF or MW ablation. The CA rate was achieved in 83.4% (78/93) of the treated tumors with RF ablation and 86.7% (91/105) in those treated with MW ablation, with no significant difference between RF and MW ablation (chi-square test , P  = 0.957).

### 2. Effects of ablation on laboratory variables

The total bilirubin (TB) levels, the AST and ALT levels were significantly elevated at 48 h in patients after both RF ablation and MW ablation compared with the baseline levels (P<0.001). The increase in the AST and ALT levels was significantly larger in MW ablation group than in RF ablation group (180.9±76.49 vs. 81.32±28.37, P<0.001 for AST, and 159.6±89.41 vs. 86.61±63.76, P<0.001 for ALT). There were no significant changes of albumin (ALB), and white blood cell (WBC) after treatment (Table 2).

**Table 2:** Effects on laboratory variables of RF and MW ablation.

Item		RF Group (n = 78)	MW Group(n = 77)	P value
AST(U/I)	Baseline	43.97±19.76	39.71±13.18	0.093
	Post ablation	143.7±46.75*	216.11±71.32*	
	Alteration	81.32±28.37	180.9±76.49	<0.001
ALT(U/I)	Baseline	43.31±15.61	38.55±23.81	0.210
	Post ablation	135.01±47.61*	208.08±91.54*	
	Alteration	86.61±63.76	159.6±89.41	<0.001
TB(µmol/l)	Baseline	18.15±3.54	18.45±6.5	0.600
	Post ablation	25.14±5.64*	31.0±10.51*	
	Alteration	6.35±5.42	11.6±7.75	<0.001
ALB(g/l)	Baseline	40.2±7.51	42.4±6.79	0.525
	Post ablation	38.55±4.62	37.0±3.41	
	Alteration	0.7±3.11	2.81±3.18	0.075
WBC(10^9^/l)	Baseline	4.31±1.31	4.5±1.35	0.255
	Post ablation	5.83±1.51	5.86±2.08	
	Alteration	1.51±1.21	0. 9±2.07	0.084

The data are expressed as mean ± SD.

AST, aspartate aminotransferase;

ALT, alanine aminotransferase.

* Compared with the baseline level, P<0.001.

### 3. Complications

Pain, fever, and asymptomatic pleural effusion were the most commonly minor complications after the treatment. Grade 1 pain (World Health Organization criteria) in the upper abdomen was observed in the intraprocedure of most patients, which was relieved immediately after the end of procedure. Twenty-three (25.9%, 23/78) patients in the RA ablation group and 46 (59.7%, 46/77) in the MW ablation group complained of post-procedural pain of Grade 1 (P = 0.018); these patient required the administration of analgesics with prescription of Nimesulide (0.1 g/d) for 3–4 days. A low-grade fever was observed after treatment in fifty-five (70.5%, 55/78) patients in the RA ablation group and 62 (80.5%, 62/77) in the MW ablation group (P = 0.589). Posttreatment asymptomatic pleural effusion was observed in nine (11.5%, 9/78) patients in the RF ablation group and 11 (14.3%, 11/77) patients in the MW ablation group (P = 0.654). There were no skin burn and tumor seeding in the study.

Major complications were observed in two of the 78 patients (2.6%) in the RF ablation group and two of the 77 (2.6%) in the MW ablation group. In the RF ablation group, major complications included persistent jaundice requiring medical therapy (n = 1) and biliary fistula requiring drainage (n = 1). In the MW ablation group, major complications included hemothorax and intrahepatic hematoma requiring drainage (n = 1) and peritoneal hemorrhage requiring blood transfusion (n = 1). The data of these complications are summarised in Table 3. There was no significant differences in major complications rate (chi-square test, P = 1.000) between treatment groups. No treatment-related death was observed in this study.

**Table 3:** Major complications of RF and MW ablation in 155 patients.

Patient	Size(cm)	Segment	Site	Approach	Complication	Treatment
1	<3	c	3	RFA	Persistent jaundice	Medical therapy
2	3–5	c	4	RFA	Biliary fistula	Drainage
3	3–5	c	7	MWA	Hemothorax, hepatic hematoma	Drainage
4	<3	s	6	MWA	Peritoneal hemorrhage	Blood transfusion

Site: c central, s superficial;

### 4. Survival

The follow-up period for the RF ablation group and MW ablation group was 26.3 months ±11.5 (range, 7.0–65.6 months) and 24.5 months ±12.9 (range, 6.0–64.0 months), respectively. During follow-up, 46 patients in the RF ablation group and 51 in the MW ablation group died. LTP was observed in eleven tumors (11.8%, 11/93) of RF ablation, and eleven tumors (10.5%, 11/105) of MW ablation. At the time of censoring, tumor DR was found in 51 (65.4%) of the patients in the RF ablation group and 62 (80.5%) of the patients in the MW ablation group, respectively. There were no significant differences in LTP (chi-square test , P  = 0.977) and DR (chi-square test , P  = 0.401) rate between treatment groups.

**Figure 3:**
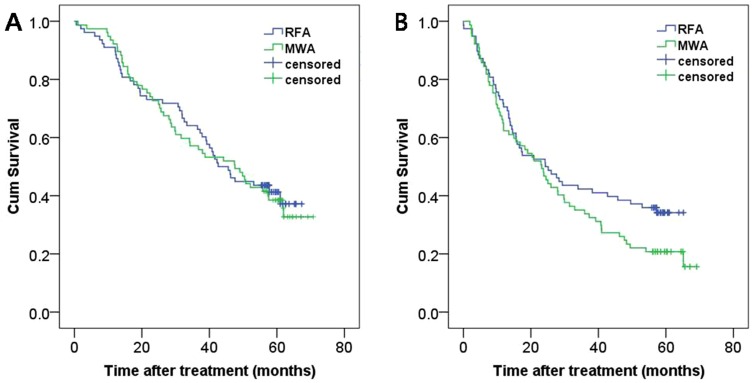
Cumulative (Cum) survival curves for patients treated with RF ablation and MW ablation. Curves show (A) overall and (B) disease-free survival.

The 1-, 3-, and 5-year overall survival rates were 91.0%, 64.1% and 41.3%, respectively, for the RF ablation group and 92.2%, 51.7%, and 38.5% for the MW ablation group. There was no significant difference between these two groups (Fig. 3a, log-rank test, P = 0.780). The 1-, 3-, and 5-year disease-free survival rates were 70.5%, 42.3%, and 34.2%, respectively, for the RF ablation group and 62.3%, 33.8%, and 20.8% for the MW ablation group. There was no significant difference between these two groups (Fig. 3b, log-rank test, P = 0.123).

### 5. Subgroup Analyses

For patients with tumors 3.0 cm or smaller, the 1-, 3-, and 5-year overall survival rates were 93.6%, 66%, and 42.6% for the RF ablation group and 100%, 80.6%, and 57.2% for the MW ablation group. There was no significant difference between these two groups (log-rank test, P = 0.067). The corresponding disease-free survival rates were 68.1%, 34%, and 27% for the RF ablation group and 72.2%, 41.7%, and 25% for the MW ablation group. There was no significant difference between these two groups (log-rank test, P = 0.849).

**Figure 4:**
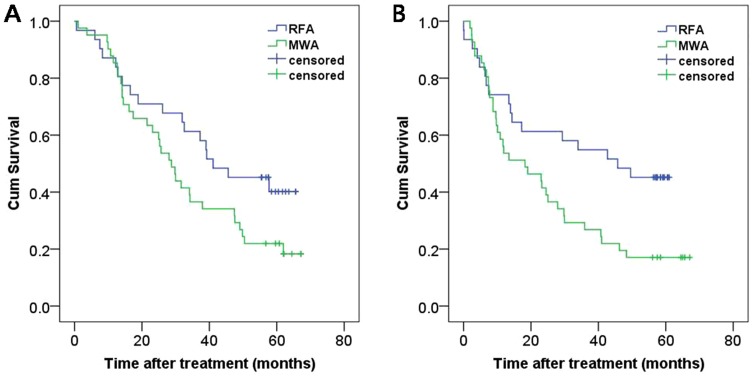
Cumulative (Cum) survival curves for subgroup analysis of patients with tumors measuring 3.1–5.0 cm. Curves show (A) overall and (B) disease-free survival for patients treated with RF ablation and MW ablation.

For patients with tumor diameters of 3.1–5.0 cm, the 1-, 3-, and 5-year overall survival rates were 87.1%, 61.3%, and 40.1% for the RF ablation group and 85.4%, 36.6%, and 22% for the MW ablation group. There was no significant difference between these two groups (Fig. 4a, log-rank test, P = 0.068). The corresponding disease-free survival rates were 74.2%, 54.8%, and 45.2% for the RF ablation group and 53.3%, 26.8%, and 17.1% for the MW ablation group. The disease-free survival curve for the RF ablation group was significantly better than that for the MW ablation group (Fig. 4b, log-rank test, P = 0.018).

## Discussion

In this study, comparison the therapeutic efficacy of percutaneous RF ablation versus MW ablation for hepatocellular carcinoma(HCC) measuring ≤5 cm in greatest diameter revealed that no significant differences in CA, LTP, DR, complication rates, and overall survival between the two groups. To our knowledge, this study represents the largest single-centre series on comparing the two modalities for the treatment of HCCs published to date. Our results broadly overlap with reports from other studies, particularly concerning the complications, CA, LTP, and the survival curves, which is comparable with other groups [11]–[14]. A randomized trial on comparing RF and MW ablation in treating Seventy-two patients with 94 HCC nodules smaller than 4 cm is worth mentioning [13]. The authors concluded that RF ablation and MW ablation have had equivalent therapeutic efficacy, complication rates, and LTP rates.

Our data showed that thermal ablation can achieve effective local tumour control, with RF ablation with CA rates of 83.4% (78/93) and LTP rates of 11.8% (11/93), and MW ablation with CA rates of 86.7%(91/105)and LTP rates of 10.5%, (11/105).

Yamashiki et al. investigated 15 explanted livers of small HCC patients who underwent MW ablation prior to liver transplantation and found CA rates of 89% [18]. In the Lu et al. study that there was no difference in local tumour control between RF and MW ablation in a group of 102 HCC patients, similar results were observed [16].

As for laboratory variables after treatment, the AST and ALT levels were significantly elevated at 48 h in patients after both RF ablation and MW ablation compared with the baseline levels (P<0.001). The increase in the AST and ALT levels was significantly larger in MW ablation group than in RF ablation group. Similar results have already been reported in our previous study [9]. The increase may be attributed to the advantages of MW ablation over RF ablation, such as higher treatment temperatures, larger ablation zones [6], [10].

As for complications, post-procedural pain was significantly more frequent after MW ablation (59.7%, 46/77) than after RF ablation (25.9%, 23/78). In the present study, we compared the performance between RF ablation and MW ablation with cooled-shaft antenna. The cooled-shaft antenna can allow MW ablation to be performed at higher power outputs (80 W) with larger ablation zones, so that the patients in the MW ablation group complained of post-procedural pain more frequent. Moreover, major complications were observed in two of the 78 patients (2.6%) in the RF ablation group and two of the 77 (2.6%) in the MW ablation group. There was no significant differences in major complications rate (P = 1.000) between the two treatment groups. Our major complications rate was close to that reported in the medical literature. An multicenter study of RF ablation of malignant liver tumors in 2320 patients reported a major complications rate of 2.2% [19]. Another multicenter study of MW ablation of liver tumors in 736 patients reported a major complications rate of 2.9% [20]. In the MW ablation group, major complications included hemothorax and intrahepatic hematoma requiring drainage (n = 1) and peritoneal hemorrhage requiring blood transfusion (n = 1). MW ablation might increase the risk of damage to the vascular structures and/or cause bleeding. In our series, the caliber of our cooled-shaft antenna was greater than that of the HiTT lateral aperture electrode, 14G vs. 15G, a difference that might account for the risk of bleeding complications we obtained.

With regard to long-term results, we found there was no significant difference between RF and MW groups of 5-year overall survival rates(91.0%,64.1% and 41.3%,vs 92.2%, 51.7%, and 38.5%). Buscarini et al. [21] reported a 5-year survival of 33% to 88 patients with 101 small HCC nodules treating by RF ablation. Dong et al. [22] reported a 1-, 3-, and 5-year survival rates of 93%, 73%, and 57% by using MW ablation in 234 patients with HCC. In the Lu et al. 's study [16], the long-term outcome of the two groups was at the same level, with 1-, 3-, and 4-year survivals of 71.7%, 37.6%, and 24.2% in the RF ablation group, and 81.6%, 50.5%, and 36.8% in the MW ablation group. For patients with tumor diameters of 3.1–5.0 cm, the 1-, 3-, and 5-year disease-free survival rates were 74.2%, 54.8%, and 45.2% for the RF ablation group, and 53.3%, 26.8%, and 17.1% for the MW ablation group. The disease-free survival curve for the RF ablation group was significantly better than that for the MW ablation group (Fig. 4b, P = 0.018). The significant difference may be attributed to the No. of tumors of MW ablation over RF ablation. In accordance with the observations of Itoh et al.'s study, multiple nodules were significant independent factors for recurrence-free survival of the disease [23].

In the past, the limitations of thermal ablation were related to long treatment duration and small ablation size. Over time, different technologies for thermal ablation have been conceived, trying to obtain better results such as local effectiveness, feasibility, and safety. Currently, there are three types of RF electrodes available commercially: an expandable needle electrode (RF2000; RadioTherapeutics MountainView, Calif; needle electrode, LeVeen, RadioTherapeutics), an internally cooled electrode (Cool-Tip RF electrode, Valleylab, Boulder, CO, USA) and an open-perfused electrode (Berchtold, Medizinelektronik, Germany) [8], [24]. MW ablation technology use frequencies ranging from 915 MHz to 9.2 GHz, and a variety of MW ablation systems using a 915-MHz or 2.45-GHz are now clinically available [25]. Studies on comparing HCCs treated by these two thermal ablative techniques have been carried out by several groups with promising results [15]–[16]. Moreover, these results come from only the comparison of one type of RF ablation generator and one ablation electrode with one type of MW ablation generator and one ablation antenna, which cannot be presumed to provide equivalent results. This is also the limitations to the study. So we think a prospective multi-institution trial with clinical validation models would be advantageous.

In conclusion, findings in this study suggest that RF ablation and MW ablation are both relatively safe procedures, with no significant differences in CA, LTP, DR, complication rates, and overall survival. Comparison of one type of ablation electrode with one type of ablation antenna cannot be presumed to provide equivalent results. Nevertheless, this result is absolutely essential for every new clinicians to make an accurate assessment of the benefits and risks of the procedure and to determine its relative and absolute indications.
